# Sensor Input Type and Location Influence Outdoor Running Terrain Classification via Deep Learning Approaches

**DOI:** 10.3390/s25196203

**Published:** 2025-10-07

**Authors:** Gabrielle Thibault, Philippe C. Dixon, David J. Pearsall

**Affiliations:** Department of Kinesiology and Physical Education, McGill University, Montreal, QC H2W 1S4, Canada

**Keywords:** convolutional neural network, inertial measurement unit, surfaces, acceleration, angular velocity, preprocessing, human activity recognition

## Abstract

**Background/Objective:** Understanding the training effect in high-level running is important for performance optimization and injury prevention. This includes awareness of how different running surface types (e.g., hard versus soft) may modify biomechanics. Recent studies have demonstrated that deep learning algorithms, such as convolutional neural networks (CNNs), can accurately classify human activity collected via body-worn sensors. To date, no study has assessed optimal signal type, sensor location, and model architecture to classify running surfaces. This study aimed to determine which combination of signal type, sensor location, and CNN architecture would yield the highest accuracy in classifying grass and asphalt surfaces using inertial measurement unit (IMU) sensors. **Methods:** Running data were collected from forty participants (27.4 years + 7.8 SD, 10.5 ± 7.3 SD years of running) with a full-body IMU system (head, sternum, pelvis, upper legs, lower legs, feet, and arms) on grass and asphalt outdoor surfaces. Performance (accuracy) for signal type (acceleration and angular velocity), sensor configuration (full body, lower body, pelvis, and feet), and CNN model architecture was tested for this specific task. Moreover, the effect of preprocessing steps (separating into running cycles and amplitude normalization) and two different data splitting protocols (leave-n-subject-out and subject-dependent split) was evaluated. **Results:** In general, acceleration signals improved classification results compared to angular velocity (3.8%). Moreover, the foot sensor configuration had the best performance-to-number of sensor ratio (95.5% accuracy). Finally, separating trials into gait cycles and not normalizing the raw signals improved accuracy by approximately 28%. **Conclusion:** This analysis sheds light on the important parameters to consider when developing machine learning classifiers in the human activity recognition field. A surface classification tool could provide useful quantitative feedback to athletes and coaches in terms of running technique effort on varied terrain surfaces, improve training personalization, prevent injuries, and improve performance.

## 1. Introduction

Running is a popular sport and recreational activity due, in part, to its accessibility and health benefits; nonetheless, running-related injuries are common. Consequently, running studies have analyzed biomechanical and physiological measures to identify relationships to common injuries, such as patellofemoral pain syndrome [1]. Running surface type has not been directly linked to injury mechanisms; however, evidence associating decreased muscle strength, a lack of neuromotor control, and irregular joint loading to injury has been presented [2]. For example, stiffer surfaces can increase the impact of training on the lower extremity joints, which can lead to injuries [3]. It is important to understand the consequences of training on different surfaces to better optimize training [4].

Earlier running studies were predominantly limited to indoor laboratory environments using treadmills or short runways, which may not adequately represent a runner’s natural overground training and competing environments [5,6,7]. Today, wearable sensors and fitness trackers make assessing training loads by surface possible. Several studies have reported outdoor measures of runners’ gait mechanics with similar accuracy as those in laboratory-controlled environments [8,9]. In particular, recent advances in wearable sensor technologies used in combination with machine learning algorithms hold the promise of accurate, multidimensional, temporal gait analysis in the real world [10]. In fact, machine learning may offer modeling of nonlinear, biological processes such as step-by-step variations due to adaptations to changes in external terrain such as orientation, obstacles, or surfaces [11]. This is relevant, as previous research has found differences in running gait variability in response to surface terrains [12,13]. These adaptations can result in changes in gait parameters such as those reported by García-Pérez et al. [6] and Van Caekenberghe et al. [7] between overground and treadmill running. Moreover, Schütte et al. [14] studied the variation in dynamic stability and loading between woodchip trails and concrete roads with a 3D accelerometer sensor located at the trunk and shank. They found a significant difference between the dynamic stability and loading of runners on both surfaces based on root mean square ratio, step/stride regularity, and sample entropy. Finally, Negi et al. [11] showed that while participants were walking, vertical acceleration provided insightful information, allowing many studies to identify gait events on different types of terrains.

Machine learning model performance is dependent on the way data are split into training and testing sets. Generally, two splitting approaches are used: subject-wise (leave-n-subject-out) and subject-dependent (randomized split; different trials from the same user may appear in the train and test sets). Worsey et al. [15] were able to classify, with an overall accuracy of 96%, an athletics track, soft sand, and hard sand using an ankle-worn accelerometer sensor and a support vector machine with ten-fold cross validation (subject-dependent); however, when the test/train split was conducted subject-wise, the results between the soft sand and the two other hard surfaces were acceptable (≥0.75 mean precision, ≥0.90 mean recall, ≥0.83 mean F1-score, ≥0.98 mean area under the curve (AUC)). No models were able to classify between the two hard surfaces (track and hard sand) with acceptable accuracy using the subject-wise split approach (<61%). To improve classification performance, calibration data, i.e., different trials from the same participant, can be included in the training dataset for each individual athlete [16]. Such calibration procedures are common in commercial wearable devices. For example, Garmin requires 15 min of data before being able to output an accurate VO2max to its user [17].

Dixon et al. [18] showed that raw acceleration data collected from two accelerometers (tibia and lower back) could classify three outdoor surfaces (concrete road, synthetic track, and woodchip trail) with high accuracies for both feature-based Gradient Booster (97.0%) and signal-based deep-learning convolutional neural network (CNN) (96.1%) models. Two main machine learning approaches are common in human activity recognition studies: feature- and signal-based deep-learning models. The latter, deep learning, was identified as the preferred method in a recent survey [19]. One of the deep-learning architectures commonly implemented is CNN, which allows multi-channel convolutions to automatically extract features from raw data and classifies the output with different activation functions instead of manually handcrafting features [20,21]. Due to their many layers, deep learning models can produce better learning capabilities [19]. CNN is the most popular model architecture for pattern recognition and has shown great performance in human activity recognition tasks by automatically and accurately detecting the patterns from different raw signals [19,22]. Preprocessing steps have been proposed in the literature to optimize the input signals for the machine learning models in running. For example, Dixon et al. [18] removed the first 2 s of their trials to only include constant running (not the acceleration/deceleration phases). They used a sliding window approach with a length of 4 s to segment the rest of the trials. Stirling et al. [23] used a similar approach but with a 300 ms window centered at heel strike, while Gao et al. [24] segmented the signal from their trials into individual strides before feeding the information to a machine learning model. Having an input signal starting at the same phase of the gait cycle makes it easier for the model to learn a pattern that may fluctuate greatly over each participant and slightly over each condition [25]. Standard preprocessing operations, such as segmentation and normalization [22,26,27] can also influence classification performance. Finally, when developing a CNN model, the different layers of operations, such as filters and pooling, allow for a gain in information on nonlinear and temporal relations of human movement. The specific combination of convolutions, pooling, rectified linear units (ReLUs), and fully connected layers will vary depending on the application [19,28] and is not well determined for human activity recognition.

Running terrain classification has been shown to be feasible using acceleration [18] and angular velocity [15] signals. To date, no research has analyzed the optimal sensor location(s) and type(s) to identify running surfaces. Moreover, the effects of standard preprocessing operations are unknown for this specific task. Thus, the primary aim of this study is to identify the optimal combination of signal type(s) and location(s) to differentiate between asphalt and grass running surfaces via a CNN deep learning approach. These two surfaces were selected as they are common surfaces that athletes run on. In addition, previous research has used those two surfaces commonly, which could facilitate comparisons with other papers. The secondary aim is to explore the effect of CNN model architectures, gait cycle segmentation, signal normalization, and two different data splitting protocols. We hypothesize that (1) the acceleration signal would outperform the angular velocity and that the lower body sensors would show the highest accuracy of classification between surfaces; and (2) gait cycle segmentation and signal amplitude normalization would improve classification performance.

## 2. Materials and Methods

### 2.1. Participants

Forty (18 female) adult casual distance runners between 18 and 50 years of age (average age was 27.4 ± 7.8 SD) were recruited for testing. Casual was defined as a minimum of 15 km per week of running. All participants were injury-free at the time of testing. The average running years of experience was 10.5 ± 7.3. This study was approved by the McGill University Ethics Board (REB File # 21-07-023).

### 2.2. Data Collection

Testing occurred at the Rutherford Reservoir, Montreal (Canada). Both running surfaces were within the same area and flat (0-degree slope), allowing us to keep the same outdoor setup for both surfaces.

**Figure 1:**
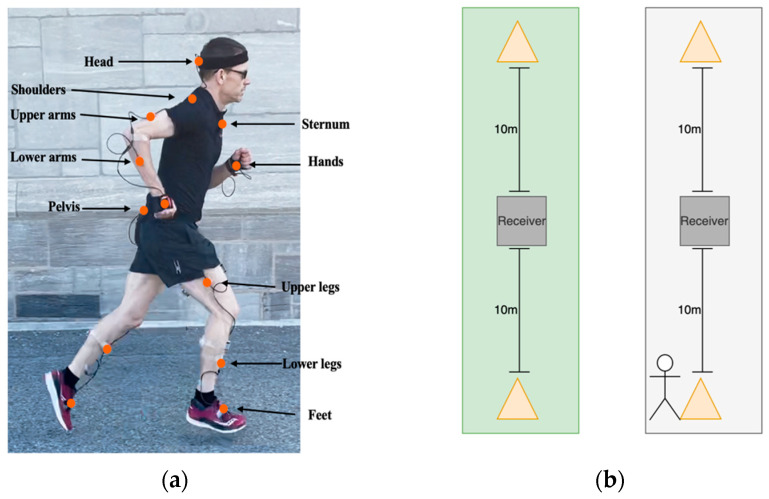
(**a**) Participant with IMU sensors. (**b**) Cone setup.

An Xsens full-body sensor system (Awinda, Xsens Technologies B.V., Enschede, The Netherlands) was positioned on the body according to manufacturer specifications and secured with medical tape [29]. The sensors were positioned on the head, sternum, pelvis, upper legs, lower legs, feet, and arms to collect 3D acceleration, angular velocity, and magnetometer data (Figure 1a). Magnetometer data were not used in this project due to potential interference from ferromagnetic substances.

The participants were given time to complete a brief warm-up and familiarize themselves with the experimental setup. All participants were asked to run on two surfaces with a consistent and comfortable submaximal pace consistent with a medium perceived effort. The trials were conducted between two cones separated by 20 m with the receiver/laptop in the middle to wirelessly transmit the data during the tests (Figure 1b). Each participant ran 80 times on each surface. The Xsens software (MVN 2021.0.1) was used to collect the IMU data and compute joint kinematics.

### 2.3. Initial Data Processing and Separation

**Figure 2:**
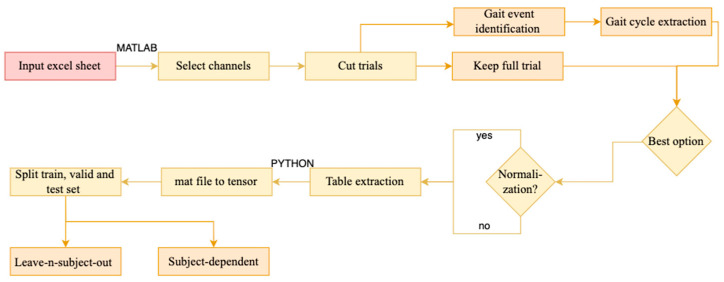
Preprocessing steps (input: red, main steps: yellow, steps with options: orange).

Preprocessing steps were performed on the raw signals using MATLAB (2017b, The Mathworks, Inc., Natick, MA, USA) via the biomechZoo toolbox (v1.3) [30] and Python 3 software (Python Software Foundation, https://www.python.org/) on Google’s Colaboratory Pro+ GPU (GPU: NVIDIA 1xTesla P100, 54.8 GB RAM). Figure 2 demonstrates the different processing steps implemented to test our study hypotheses.

Two different signal separation approaches were compared using the lower body sensor combination. The first was a four-second section of the trials without the acceleration and deceleration phases (beginning and end of trial). The second method extracted gait cycles from trials and used them as the inputs for the model [31]. Heel strike was identified using the first local minimum between each maximum knee flexion peak [30]. A total of 43,803 running gait cycles were collected, with 49.4% of them on grass and 50.6% on asphalt. During the trials, if a sensor fell, a note was taken on the participant sheet, and that trial was eliminated from the rest of the analysis.

The impact of signal amplitude normalization on the input signals was also tested. More specifically, all signals were divided by the maximum value for their individual trial and channel.

Finally, processed files were loaded into Python and converted into the correct tensor shape: #trials X #frames X #channels, as per the recommendation of Chollet [32].

**Figure 3:**
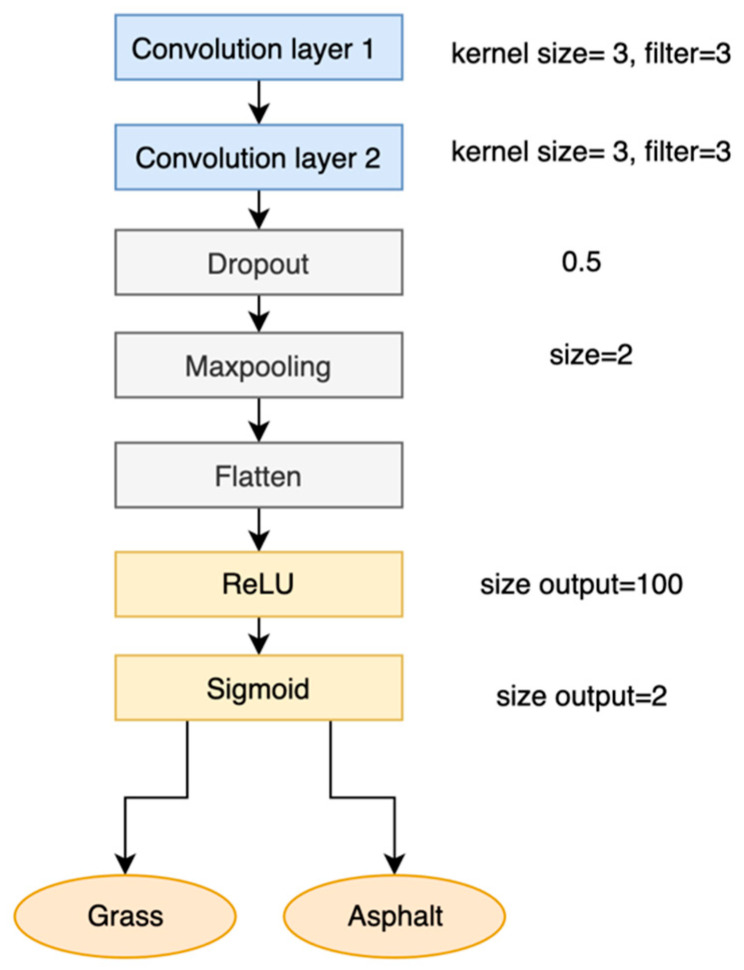
Model for preliminary testing (convolutional layers: blue, regularization, dimensionality reduction, and preparation for classification: gray, activation functions: yellow, outputs: orange).

Four sensor combinations were tested for the acceleration and angular velocity signals (Table 1) using a basic model (Figure 3).

Only 12 out of the 17 sensors were used in the analysis of the preprocessing steps. The sensor combinations were determined based on initial testing. After obtaining preliminary results showing a better performance of the model with only the lower body, it was concluded that a deeper analysis would be conducted on the individual sensors of that body segment.

### 2.4. CNN Model Architecture

First, starting with a basic model (Figure 3), one to four convolutional layers were tested to determine the optimal general CNN architecture [32]. Then, three different optimizers (Adam, RMSprop, and SGD) and different batch sizes were evaluated. Second, the model hyperparameters were tuned using KerasTuner (https://keras.io/keras_tuner/ accessed on 1 June 2019) and a callback function for early stopping was implemented (using the validation loss with a patience of 50). The learning rate, number of filters, kernel size, dropout, and regularization ratio were tuned. A regularization parameter (Table 2) was also initially tested with KerasTuner but then removed due to lower performance on the validation accuracy when included in the model.

**Figure 4:**
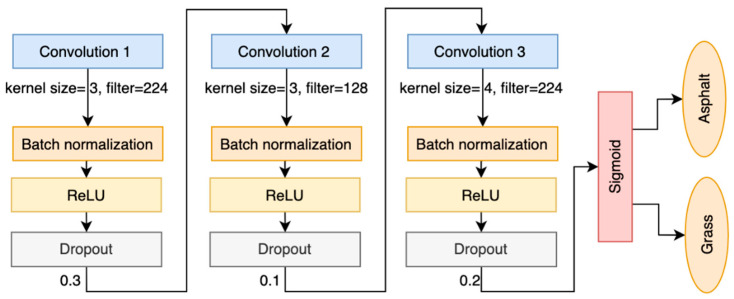
Convolutional neural network architecture (convolutional layers: blue, batch normalization: orange, ReLU: yellow, dropout: gray, activation function: red, outputs: orange).

Batch normalization was used in the final CNN model to stabilize and speed up the training phase (Figure 4).

The following steps were conducted using the optimal combination of sensors found in the previous section.

### 2.5. Train, Validation, and Test Split

Two different splitting approaches were tested—subject-wise and random (subject-dependent). The subject-wise split (leave-n-subject-out) separates the datasets with different participants in train, test, and validation sets (inter-subject split). The subject-dependent split shuffled all trials before separating the three dataset subsections (intra-subject split). Thus, in the latter approach, different trials from the same participant may appear in the training, testing, and validation sets.

### 2.6. Model Performance Analysis

The CNN models were developed, trained, and tested on Google’s Colaboratory Pro+ GPU (GPU: 1x Tesla P100, 54.8 GB RAM). All the steps tested for the preprocessing of this analysis can be found in a diagram (Figure 2). Both the acceleration and angular velocity were tested with the final optimized model using the best sensor combination. Precision, recall, F1-score, and accuracy were evaluated for both surfaces.

## 3. Results

### 3.1. Effect of Preprocessing Steps and Sensor Combination

For the preprocessing steps tested, separating the trials into gait cycles (accuracy for lower body combination sensors: 91.8% vs. 63.6%) and not normalizing the raw signals (accuracy for lower body combination sensors: 91.8% vs. 89.2%) improved performance for both the acceleration and the angular velocity combinations.

To determine the optimal sensor signal type and location, the four combinations mentioned in Table 1 were tested. In general, the acceleration combinations provided better results. The highest accuracy with the lowest number of sensors was obtained at the foot (92%). These results (effect of preprocessing and sensor combination testing) were obtained with the baseline CNN model (Figure 3).

### 3.2. Effect of Network Optimization

Based on the results of Section 3.1, the optimized CNN model was deployed using trials cut into gait cycles and non-normalized (raw) signals.

**Figure 5:**
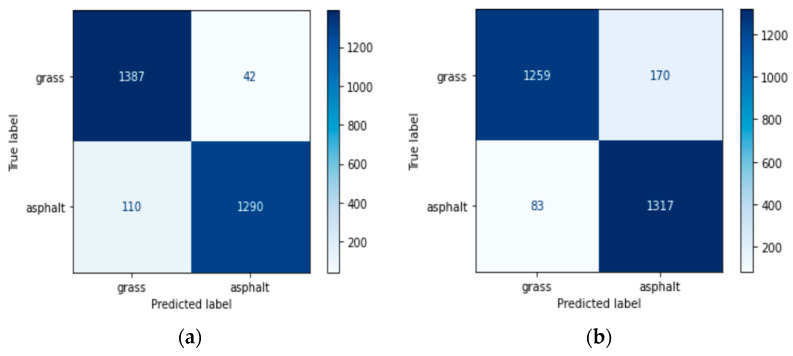
Confusion matrix with the final model using (**a**) acceleration and (**b**) angular velocity signals.

In Table 3 and Figure 5, the results for the final CNN model can be found for both the acceleration and angular velocity using the foot sensor. The acceleration gives better results for all sensor combinations.

Finally, Table 4 shows the effect of including different trials from the same subjects in the train and test sets on classification accuracy between the two different surfaces. The subject-dependent approach gives better results. Models benefited from “seeing” previous trials from the same participant (performance of the model was improved).

## 4. Discussion

### 4.1. Summary

The main purpose of this study was to determine the effect of signal type(s), location(s), preprocessing steps, and data splitting on the classification of grass and asphalt surfaces via a deep learning model during running. The foot sensor acceleration was found to be the best location/type of sensor to classify between the running surfaces.

Two different preprocessing steps and four different sensor combinations were tested for the acceleration and angular velocity signals. In general, the acceleration signals produced better results than the angular velocity for accuracy, precision, recall, and F1-score. Two different splitting approaches, separating the dataset into training, validation, and testing sets, were also compared. The maximum classification accuracy was achieved using a subject-dependent split approach. One of the strengths of this study was the sample size. Previous work had fewer participants and fewer trials [33].

### 4.2. Preprocessing

Two preprocessing steps were tested to ensure the highest classification performance. First, separating the trials into gait cycles increased the accuracy of prediction, giving a simpler and more repetitive pattern for the model to learn from. The surfaces tested were very similar, which increases the difficulty of finding specific patterns for each task. This could explain why separating the trials by gait cycle had such a significant impact on the performance of the model. Cutting the full trials to four seconds, to remove the acceleration/deceleration phases, provided us with input signals starting at different phases of the gait cycle. This makes it harder for the model to learn a pattern that fluctuates greatly over each participant and slightly over each condition [25]. It is also a common preprocessing step to standardize the input signals by bringing them to a common scale (amplitude normalization) [26]. Scaling the input signals brings all data to the same number of intervals, which makes it easier for the model to compare and associate different inputs. Here, we analyzed the impact of max-normalizing and found that, for this specific task, it did not have a major impact on accuracy (decreased < 2%); however, the speed of training decreased slightly when removing this preprocessing step. There might have been information in the signal related to amplitude that helped distinguish the surfaces. Hence, normalization of data was deemed unnecessary for this project.

### 4.3. Sensor Location and Count

First, the all-body sensor configuration and lower-body sensor configuration were compared to determine the impact of the upper-body sensors. It was concluded that the upper body sensors did not provide as much information to differentiate between surfaces as the lower body sensors (78% and 92% for acceleration, respectively). Second, the lower body sensors were individually tested. The pelvis sensor produced the lowest results, and the foot sensors produced the highest results (92% and 79% for acceleration, respectively). The foot sensors are the closest sensors to the surface tested, which makes them more susceptible to small movement caused by terrain variations. Our results are in agreement with Dixon et al. [18], who also obtained better results with the tibia, compared to the lower back sensor, using a CNN model for running surface classification.

### 4.4. Splitting Approaches

Two different splitting approaches were tested: subject-wise (inter-subject) and a random split (intra-subject). Similarly to previous research, the results using the subject-dependent split were significantly better [34]. Worsey et al. [15] compared an athletics track, soft sand, and hard sand using an ankle accelerometer and obtained acceptable results using a subject-wise approach wearing an ankle accelerometer (≥0.75 mean precision, ≥0.90 mean recall, ≥0.83 mean F1-score, ≥0.98 mean area under the precision–recall curves across all models). They saw significant improvement when some calibration data were given to the model before evaluating it on the test set (subject-dependent). Worsey et al. [15] obtained <61% for the athlete-independent method and <97% for the athlete-dependent method. Using the subject-wise protocol, our model classified grass and asphalt with an overall accuracy of 81% with the acceleration-foot sensors. The subject-dependent splitting protocol yielded high-performance classification results for this project and Worsey et al.’s [15] study (95.5% and <97%, respectively). Even using the subject-dependent approach, Worsey et al. [15] observed that the performance was lower when classifying between the two hard surfaces (athletics track and hard sand). In this study, the surfaces tested were very similar (e.g., the *p*-value for lower body sensors’ vertical acceleration was between 0.9627 and 1.000). The more similar the patterns that we want to classify, the harder it is for the model to find differences between participants without calibration data. This could easily be incorporated into the device workflow by asking the users to run with the wearable device for a certain amount of time before receiving “type of surfaces” feedback to allow it to calibrate to the specific runner.

### 4.5. Limitations

Despite the strong performance of the models, there are limitations within the current study to discuss. First, data collection did not fully represent a complete running session where an athlete would accelerate/decelerate and switch between surfaces. Adding more variations in the input signals would have made it harder for the model to learn the specific patterns for each surface but would have made it more representative of real-world situations. Second, this study only included two common running surfaces (grass and asphalt); however, numerous other surfaces may be encountered in real-world running (e.g., snow/ice, sand, gravel, and trails). Our work provides insight into which sensor combinations are most effective for distinguishing between grass and asphalt and could potentially be generalized to other surfaces. Since the model has not been trained on these additional surfaces, accurate predictions would require collecting sufficient data from another large participant pool (e.g., 50+ runners across multiple surfaces). The more similar the surfaces, the more difficult they are to classify, as the algorithm has fewer differences in the output signals to learn from. Third, this model has not been tested on trials where the runners were transitioning between different surfaces. Further research is needed in that area. Finally, this research may not generalize injured people or runners younger than 18 or older than 50 years old.

## 5. Conclusions

This study showed the significant benefit of trial segmentation into gait cycles before being fed into the CNN model. The foot acceleration signals were found to be the best combination for a performance-number of sensor ratio optimization. Surface classification can be achieved with acceleration signals alone and could be integrated into wearable devices such as sports watches to improve the accuracy of the training effect outputted from those technologies and, hence, give better feedback for athletes to improve performance and reduce injury rates.

## Figures and Tables

**Table 1:** Sensor combinations tested for acceleration and angular velocity.

Number of Triaxial Sensors	Combinations
12	Full body (head, left and right shoulder, left and right upper arm, left and right hands, pelvis, left and right lower leg, and left and right feet)
5	Lower body (pelvis, left and right lower leg, and left and right feet)
1	Pelvis
2	Feet (left and right feet)

**Table 2:** Tuning the CNN model.

Hyperparameters	Options
Epochs	Using callback for early stop (patience 50)
Batch size	50, 100, 200, and 300
Optimization function	Adam, RMSprop, and SGD
Learning rate	From 0.0001 to 0.01
**Model architecture**	**Options**
Number of convolutional layers	From 1 to 4
Filter number	From 32 to 256 (step 32)
Kernel size	From 3 to 5 (step 1)
Dropouts	From 0 to 0.5 (step 0.1)
Regularization	L1, L2, and L1_L2

**Table 3:** Performance analysis of the final model. Epoch = 500 (callback); batch size = 200.

Sensor Signal Type	Surfaces	Precision	Recall	F1-Score	Accuracy (%)
3D	Grass	0.97	0.95	0.95	95.71
Acceleration	Asphalt	0.97	0.92	0.94	
3D Angular velocity	Grass	0.94	0.88	0.91	91.91
	Asphalt	0.89	0.94	0.91	

**Table 4:** Accuracies (%) for different split protocols. Epoch = 500 (callback), batch size = 200.

	Split Protocol	Accuracy
Acceleration	Leave-n-subject-out	82.32
	Subject-dependent	95.71
Angular velocity	Leave-n-subject-out	57.58
	Subject-dependent	91.91

## Data Availability

The datasets presented in this article are not readily available because it was not part of the Ethics Board protocol to share the collected data.

## Abbreviations

The following abbreviations are used in this manuscript:

CNNs	convolutional neural networks
IMU	inertial measurement unit
AUC	area under the curve
ReLUs	rectified linear units
